# The Production of Nitric Oxide, IL-6, and TNF-Alpha in Palmitate-Stimulated PBMNCs Is Enhanced through Hyperglycemia in Diabetes

**DOI:** 10.1155/2014/479587

**Published:** 2014-04-06

**Authors:** Caroline Maria Oliveira Volpe, Luana Farnese Machado Abreu, Pollyanna Stephanie Gomes, Raquel Miranda Gonzaga, Clara Araújo Veloso, José Augusto Nogueira-Machado

**Affiliations:** ^1^Núcleo de Pós-Graduação e Pesquisa (NPGP), Hospital Santa Casa de Belo Horizonte, Domingos Vieira 590, Santa Efigênia, 30150-240 Belo Horizonte, MG, Brazil; ^2^Centro Universitário de Belo Horizonte (UniBH), Professor Mário Werneck, 1685 Estoril, 30455-610 Belo Horizonte, MG, Brazil

## Abstract

We examined nitric oxide (NO), IL-6, and TNF-*α* secretion from cultured palmitate-stimulated PBMNCs or in the plasma from type 2 diabetes mellitus (T2MD) patients or nondiabetic (ND) controls. Free fatty acids (FFA) have been suggested to induce chronic low-grade inflammation, activate the innate immune system, and cause deleterious effects on vascular cells and other tissues through inflammatory processes. The levels of NO, IL-6, TNF-*α*, and MDA were higher in supernatant of palmitate stimulated blood cells (PBMNC) or from plasma from patients. The results obtained in the present study demonstrated that hyperglycemia in diabetes exacerbates *in vitro* inflammatory responses in PBMNCs stimulated with high levels of SFA (palmitate). These results suggest that hyperglycemia primes PBMNCs for NO, IL-6, and TNF-alpha secretion under *in vitro* FFA stimulation are associated with the secretion of inflammatory biomarkers in diabetes. A combined therapy targeting signaling pathways activated by hyperglycemia in conjunction with simultaneous control of hyperglycemia and hypertriglyceridemia would be suggested for controlling the progress of diabetic complications.

## 1. Introduction

Circulating free fatty acids (FFAs) are elevated in patients with type 2 diabetes mellitus (T2DM), obesity, metabolic syndrome, and dyslipidemia [1–4]. FFAs represent a complex group of structurally variable molecules stored in the body as triglycerides and released through lipolysis [3, 5]. FFAs are classified according to the carbon chain length in short-, medium-, and long-chain fatty acids, the presence or absence of double bonds as saturated (SFA) and unsaturated fatty acids, respectively, and the number of double bonds as mono- or polyunsaturated (PUFA) [6, 7]. The effect of FFA on cellular signaling pathways depends on the chemical structure. It has been reported that chronic exposure to SFA increases oxidative stress and inflammation, leading to the development of cardiovascular diseases and insulin resistance [8–12].

Oxidative stress, reflecting an imbalance between prooxidant and antioxidant effectors, plays an important role in diabetic vascular complications [13]. Superoxide, nitric oxide, and lipid peroxidation are indicators of oxidative stress in the body. Despite the number of studies concerning FFA-induced superoxide overproduction [14–22], there are few reports concerning FFA-induced nitric oxide (NO) production. NO is a highly diffusible and unstable gas that acts as a modulator of vascular tone, glucose transport in skeletal muscle cells and adipocytes, blood flow, force generation in skeletal muscle, cytotoxicity, and inflammation [23–26].

FFA also regulates the immune system through interactions with specific cell surface receptors, such as Toll-like receptors (TLR) and G-protein-coupled receptors (GPCR), thereby activating NF-kappaB and c-Jun amino-terminal kinase (JNK) pathways, which stimulate the secretion of proinflammatory cytokines (IL-1beta, IL-6 and TNF-alpha) and chemokines [27–30].

It is well known the effects of hyperglycemia and hyperlipidemia on peripheral blood mononuclear cells (PBMNCs) by activation of NADPH oxidase system leading to reactive oxygen species production, TLR expression, enhancing NF-kappaB activity, and inducing proinflammatory cytokines, chemokines, and circulating adhesion molecules secretion [8, 21, 31–41].

Thus, elevated plasma FFA levels act as inflammatory inducers, which potentially contribute to vascular disorders [27–30, 42, 43]. Thus, the aim of the present study was to investigate the* in vitro* effects of palmitate (C16:0), the major SFA in plasma [44, 45], on the modulation of oxidative stress and inflammation in T2DM patients. Nitric oxide, with or without palmitate induction, was quantified and correlated with proinflammatory cytokines secreted in the cultured supernatant of PBMNCs from type 2 diabetes patients. The association among plasmatic triglycerides, NO, proinflammatory cytokines (IL-6 and TNF-alpha), and oxidative stress (malondialdehyde) is discussed.

## 2. Material and Methods

This study was approved through the Ethical Committee of Santa Casa Hospital (Belo Horizonte-MG, Brazil) and written informed consent was obtained from all participants prior to the study.

### 2.1. Subjects

T2DM patients (*n* = 29), diagnosed according to the criteria of the American Diabetes Association [46], and nondiabetic controls (*n* = 16), ranging from 45 to 70 years of age, were recruited from the Endocrinology Department of Santa Casa Hospital. Type 2 DM patients were treated with statins and beta-blockers in addition to hypoglycemic drugs. Prior to the study, all volunteers received complete physical examinations, and detailed evaluations of medical histories and laboratory analyses were performed (Table 1). Pregnant women and individuals suffering from alcoholism, infection, inflammation, dementia, or malignant diseases and smoking addictions were excluded from this study.

### 2.2. Preparation of Fatty Acids

Palmitate and low-endotoxin bovine serum albumin (BSA, FFA-free) were purchased from Sigma-Aldrich Co. FFA was dissolved in 0.1 M NaOH at 70°C and subsequently complexed with 10% BSA at 55°C for 10 min to obtain a final FFA concentration of 500 *μ*M (molar ratio 2.4 : 1) [42, 47]. A 10 mM fatty acid-albumin complex stock solution and a 0.5 *μ*M BSA control solution were freshly prepared, filtrated, and diluted prior to each experiment.

### 2.3. Preparation of Peripheral Blood Mononuclear Cells

PBMNCs were purified from 10.0 mL of heparinized venous blood, using a Ficoll-Hypaque gradient as previously described [48], with slight modifications. The trypan blue exclusion test showed that the cell viability in all samples was >95%.

### 2.4. Preparation of Plasma

EDTA venous blood samples were collected using a standard venipuncture technique. The plasma was obtained through centrifugation (200 g for 15 min, at room temperature), and the samples were stored at −80°C until further analysis. Subsequent analyses were performed within 3 months from the day of storage.

### 2.5. Quantification of Proinflammatory Cytokines and NO in Supernatant of PBMNCs

Aliquots (100 *μ*L) of a PBMNC suspension (1 × 10^6^/mL) from T2DM patients and ND controls in Dulbecco's modified Eagle's medium (DMEM) supplemented with 10% fetal bovine serum (FBS) were incubated in the presence or absence of BSA (0.5 *μ*M) or palmitate (500 *μ*M) for 72 hours at 37°C under 5% CO_2_. The final volume was adjusted to 300 *μ*L in DMEM supplemented with 10% FBS. After incubation, the cells were centrifuged and the supernatant was collected. The interleukin-6 (IL-6 human EIA Kit—Enzo Life Sciences, Inc., New York, USA) and tumor necrosis factor-alpha (TNF-*α* human EIA Kit—Enzo Life Sciences, Inc., New York, USA) concentrations were determined through enzyme-linked immunosorbent assay (ELISA). Because NO is unstable, the quantitative of NO was indirectly determined based on the detection of the blood nitrite and nitrate levels. The NO concentration was measured using the Total Nitric Oxide Assay Kit (Assay Designs, Enzo Life Sciences, Inc., New York, USA).

### 2.6. Quantification of NO, MDA, and Proinflammatory Cytokines in Plasma

The plasma levels of NO, IL-6, and TNF-alpha were determined as described above. The plasma MDA concentration was measured using the TBARS Assay Kit (ZeptoMetrix Corp., New York, USA) according to the manufacturer's instructions.

### 2.7. Statistical Analyses

The values are presented as the means ± standard deviation (SD). The nonparametric Kolmogorov-Smirnov test was used to assess the normal distribution of the continuous variables. Comparisons between groups were performed using unpaired Student's* t*-tests. Within-group correlations were performed using Pearson's *r* correlation. All analyses were considered significant at *P* values < 0.05 using Origin 6.0 software (Microcal Software Inc., Northampton, MA, USA).

## 3. Result

### 3.1. PBMNCs from T2DM Patients Are More Sensitive to Palmitate Stimulation Than the Cells from ND Controls

**Figure 1:**
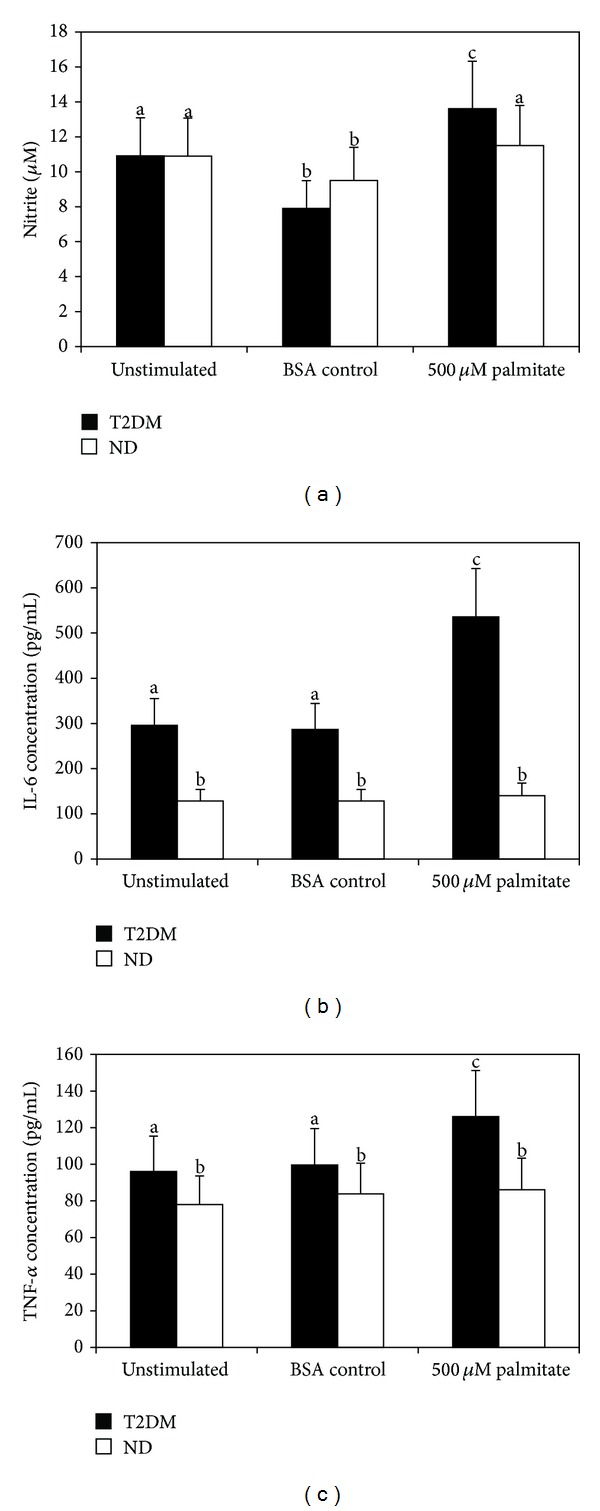
Palmitate induces NO, IL-6, and TNF-alpha secretion in peripheral blood mononuclear cells (PBMNC) from patients with type 2 diabetes. (a) Nitrite production; (b) IL-6 production; (c) TNF-alpha production. Different letters denote significance at *P* < 0.05 using Student's* t*-test. *n* = 10 for each group.

As depicted in Figure 1, palmitate activated the secretion of NO, IL-6, and TNF-alpha in PBMNCs from T2DM patients compared with those from ND controls (*P* < 0.05). The results of the induced effect of palmitate on PBMNCs from T2DM patients and ND controls, expressed as the means ± SD, were NO, 11.5 ± 1.3 and 13.6 ± 2.2; IL-6, 86.1 ± 14.1 and 126.0 ± 29.0; and TNF-alpha, 140.0 ± 28.1 and 535.8 ± 115, respectively. The results shown in Figure 1 also demonstrated that PBMNCs from T2DM patients secreted significantly (*P* < 0.05) higher amounts of IL-6 (256.7 ± 81.1) and TNF-alpha (96.1 ± 17.5) compared with the cells from ND controls (IL-6: 128.3 ± 32.3, TNF-alpha: 78.0 ± 13.6). No difference (*P* > 0.05) was observed in NO production in PBMNCs from T2DM patients (10.9 ± 1.7) and ND controls (10.9 ± 1.2) without stimulation.

The production of NO and proinflammatory cytokines was not altered in the presence of BSA (*P* > 0.05) in T2DM patients and ND controls: NO, 11.5 ± 1.3 and 13.6 ± 2.2; IL-6, 86.1 ± 14.1 and 126.0 ± 29.0; and TNF-alpha, 140.0 ± 28.1 and 535.8 ± 115, respectively.

### 3.2. Palmitate-Induced NO and IL-6 Production in PBMNCs Are Associated in T2DM Patients, but Not in ND Controls

**Figure 2:**
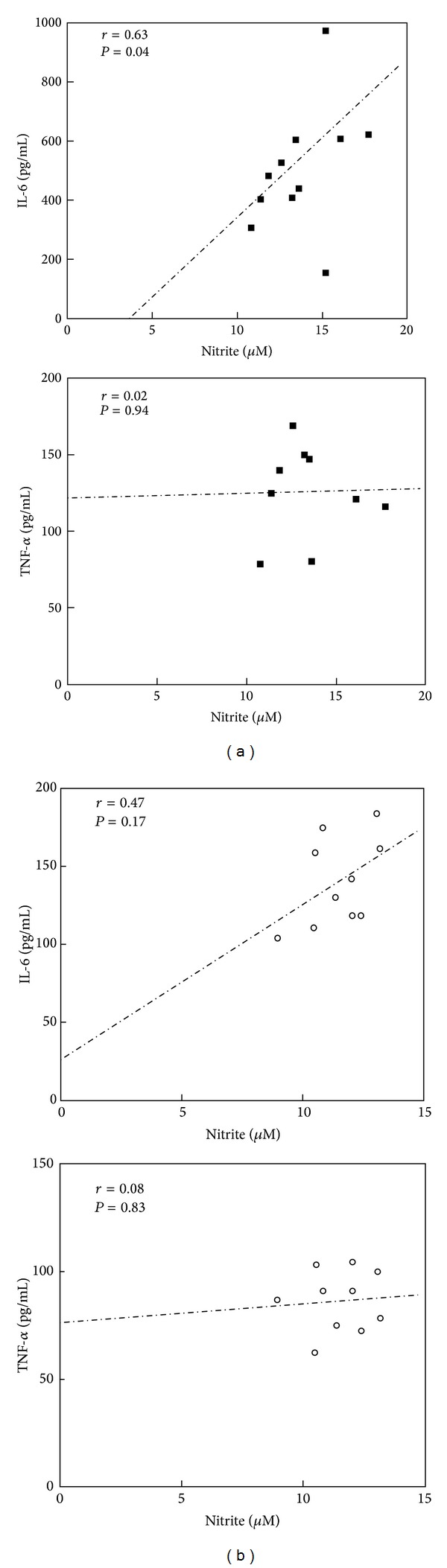
Pearson's correlation coefficients between NO and proinflammatory cytokines in PMBNCs from T2DM patients (a) and nondiabetic controls (b) after stimulation with palmitate. *n* = 10 for each group.

Figure 2 shows the Pearson's correlations between the levels of NO, IL-6, and TNF-alpha in PBMNCs from T2DM patients and ND controls after palmitate stimulation. The correlation between NO and IL-6 were significantly strong in stimulated PBMNCs from T2DM patients (*r* = 0.63, *P* = 0.04) and moderate in PBMNCs from ND (*r* = 0.47, *P* = 0.17). No correlation was observed between NO and TNF-alpha in PBMNCs from T2DM patients and ND controls.

### 3.3. The Plasma MDA and Proinflammatory Cytokine (IL-6 and TNF-Alpha) Concentrations Are Elevated in T2DM

Table 2 shows that T2DM patients had enhanced plasma concentrations of MDA, IL-6, and TNF-alpha compared with ND (*P* < 0.05). No difference was observed in NO levels between T2DM patients and ND (*P* > 0.05). The results, expressed as the means ± SD, were MDA, 14.5 ± 3.5 and 8.7 ± 3.3; IL-6, 119.1 ± 23.3 and 97.6 ± 13.5; TNF-alpha, 78.7 ± 32.7 and 58.5 ± 29.5; NO, 53.5 ± 12.9 and 51.13 ± 8.7, for T2DM patients and ND controls, respectively.

### 3.4. Plasmatic Nitric Oxide Levels Correlate with MDA and IL-6 Levels in the Plasma from T2DM Patients

**Figure 3:**
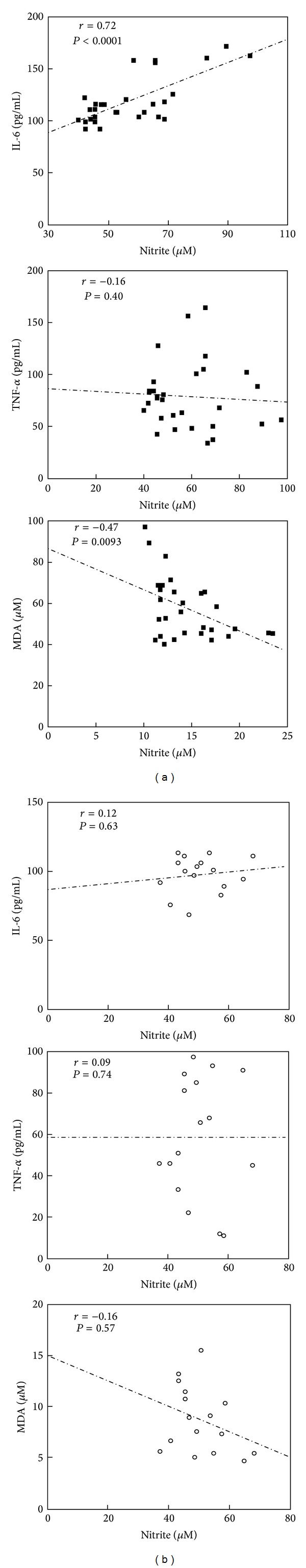
Pearson's correlation coefficients between nitric oxide and proinflammatory cytokines and MDA in the plasma from T2DM patients (a) and nondiabetic controls (b). *n* = 29 for T2DM patients and 16 for nondiabetic controls.

Correlations between the levels of NO and IL-6 and TNF-alpha and MDA are shown in Figure 3. Strong positive correlation was observed between NO and IL-6 in T2DM patients (*r* = 0.72, *P* < 0.0001). The results also demonstrated a significantly negative correlation between NO and MDA in T2DM patients (*r* = −0.47, *P* = 0.0093).

### 3.5. Plasmatic Triglyceride Levels in T2DM Patients Correlate with the Plasma Levels of MDA, IL-6, and TNF-Alpha

**Figure 4:**
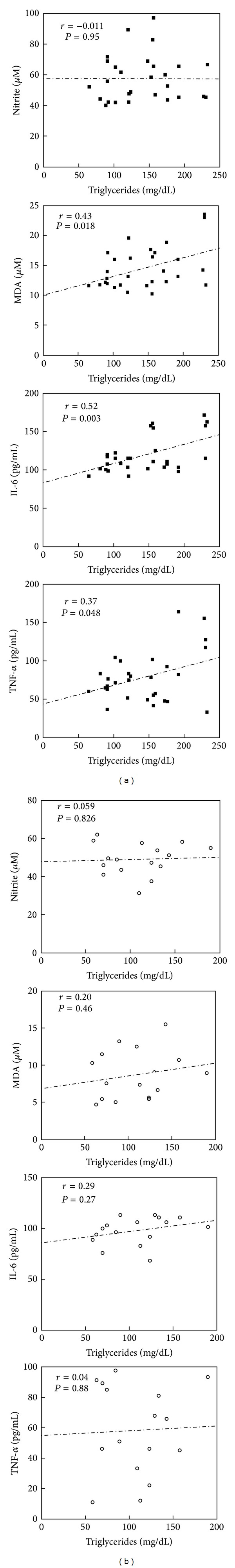
Pearson's correlation coefficients between triglycerides and proinflammatory cytokines and MDA in the plasma of T2DM patients (a) and nondiabetic controls (b). *n* = 29 for T2DM patients and 16 for nondiabetic controls.

Figure 4 shows the Pearson's correlations between the levels of triglyceride and NO and IL-6 and TNF-alpha in the plasma from T2DM patients and ND. The triglyceride levels were positively correlated with MDA (*r* = 0.43, *P* = 0.018), IL-6 (*r* = 0.52, *P* = 0.003), and TNF-alpha (*r* = 0.37, *P* = 0.048) in the plasma of T2DM patients.

### 3.6. Plasmatic Glucose Levels in T2DM Patients Correlate with the Plasma Levels of Triglycerides, MDA, IL-6, and TNF-Alpha

**Figure 5:**
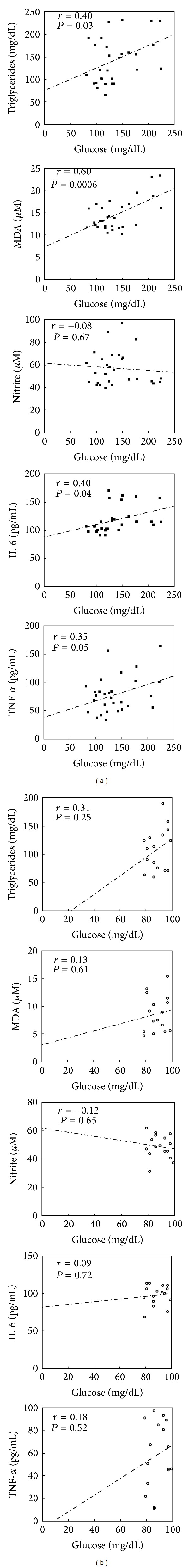
Pearson's correlation coefficients between glucose and triglycerides, MDA, nitric oxide, and proinflammatory cytokines in plasma of T2DM (a) patients and nondiabetic controls (b). *n* = 29 for T2DM and 16 for nondiabetic controls.

Figure 5 shows the Pearson's correlations between the levels of glucose and triglycerides, NO, MDA, and proinflammatory cytokines levels in the plasma from T2DM patients and ND controls. The glucose levels were positively correlated with triglycerides (*r* = 0.40, *P* = 0.03), MDA (*r* = 0.60, *P* = 0.0006), IL-6 (*r* = 0.40, *P* = 0.04), and TNF-alpha (*r* = 0.35, *P* = 0.05) in the plasma of T2DM patients.

## 4. Discussion

The results obtained in the present study showed that hyperglycemia in diabetes primes PBMNCs* in vivo*, inducing the* in vitro* upregulation of NO and proinflammatory cytokines in cells stimulated with palmitate. The plasmitic evaluation demonstrated greater levels of triglycerides, MDA, IL-6, and TNF-alpha in T2DM patients compared with ND. No difference was observed in the NO plasma levels between T2DM patients and ND. In addition, the results of this study revealed that the levels of NO were correlated with MDA and IL-6, and levels of triglycerides were correlated with MDA, IL-6, and TNF-alpha in the plasma from T2DM patients.

Diabetes is a multifactorial disease characterized by hyperglycemia and hyperlipidemia, which are important risk factors for endothelial dysfunction resulting in cardiovascular events [49]. FFAs, particularly SFA, have been shown to induce a proinflammatory profile associated with obesity, T2DM, insulin resistance, and dyslipidemia [4, 8–11]. The results presented herein show the inflammatory effects of the saturated fatty acid palmitate on PBMNCs from T2DM patients but not in cells from ND (Figure 1), suggesting that hyperglycemia plays a role in palmitate-induced inflammation. Studies have shown that the combined effect of high glucose and FFA levels in human monocytes modulate macrophage proliferation involving glucose-dependent oxidation of LDL, potentiate cytotoxic effects via superoxide overproduction, and amplify inflammation via TLR [21, 50, 51]. However, Tripathy et al. [32] demonstrated that an increase in FFA concentration induces oxidative stress and inflammation in human leukocytes from ND subjects. These discrepancies might be associated with differences in the experimental protocols.

The inflammatory changes observed in the presence of palmitate could be associated with NF-kappaB activation [21, 28, 32, 52–55]. NF-kappaB is a key mediator that regulates immune and inflammatory responses and modulates multiple proinflammatory target genes in endothelial cells, vascular smooth muscle cells, and macrophages [56]. The activation of NF-kappaB leads to the increased production of adhesion molecules, leukocyte-attracting chemokines, various inflammatory cytokines, including TNF-alpha and IL-6, and NO through iNOS expression [57–60].

NO has anti- or proinflammatory properties [61]. NO plays an important role in vascular homeostasis, and in immune cells, NO regulates antimicrobial and antitumor activities, although excess NO production might cause tissue damage and is associated with acute and chronic inflammation [56, 62]. Nitric oxide synthase (NOS) synthesizes NO from L-arginine using NADPH and oxygen as cosubstrates [63]. Three isoforms of NO synthase have been described: neuronal (nNOS or NOS 1), inducible (iNOS or NOS 2), and endothelial (eNOS or NOS 3) [64]. Activated macrophages and neutrophils produce large amounts of NO through iNOS activity [65, 66]. The results of this study demonstrated increased NO production and a positive correlation between NO and IL-6 levels in palmitate-stimulated PBMNCs from T2DM patients, suggesting that iNOS expression can be elevated through palmitate-induced proinflammatory cytokine secretion. No differences were observed in the cells from ND controls (Figures 1 and 2). Unbound palmitic acid treatment increased NO production in skeletal muscle [67]. However, in endothelial cells, FFA induced the inhibition of eNOS, thereby attenuating NO production [68–71].

To evaluate* in vivo *inflammation, we quantified the plasma levels of NO, the oxidative stress biomarker (MDA), and proinflammatory cytokines (IL-6 and TNF-alpha) in T2DM patients and ND controls. Consistent with other studies [72–93], the results of the present study demonstrated elevated levels of IL-6 and TNF-alpha, reflecting the activation of innate immune cells, and high levels of MDA, indicating the presence of oxidative stress in T2DM patients compared with ND controls. Diabetic conditions (hyperglycemia and hyperlipidemia) increase proinflammatory and oxidative stress levels, culminating in endothelium dysfunction [1, 27, 42, 56, 90, 94, 95]. Oxidative stress reduced NO production through eNOS [56], and the increased levels of superoxide could react with NO to produce peroxynitrite, a highly toxic product [23, 96]. Peroxynitrite nitrates the tyrosine residues in a number of proteins and modulates their functions [97, 98]. The results in the present study did not show any differences in the plasma NO levels between the studied groups (Table 2). However, we observed a negative association between NO and MDA levels in the plasma from T2DM patients, suggesting that increased oxidative stress could affect NO biodisponibility, leading to endothelial dysfunction in diabetes (Figure 3).

The results obtained in the present study also demonstrated high levels of triglycerides in the plasma from T2DM patients compared with ND controls (Table 1). FFAs are stored in the body in the form of triglycerides and are released into tissues through lipolysis, a process regulated through insulin [99]. Impaired insulin signaling increases lipolysis, resulting in increased FFA levels [100, 101]. The results of the present study showed that triglycerides levels are positively associated with the MDA, IL-6, and TNF-alpha levels in the plasma from T2DM patients, but this correlation was not observed in the plasma from ND controls. No correlation was observed between triglycerides and NO in the plasma from the studied groups (Figure 4). Glucose levels are positively correlated with the triglycerides, MDA, IL-6, and TNF-alpha levels in the plasma from T2DM patients, but not in the plasma from ND controls (Figure 5).

Accumulating evidence has shown that the regulation of dyslipidemia is of equal importance for the regulation of hyperglycemia and hypertension in the care of patients with T2DM. Hyperlipidemia represents a major risk factor for the development of vascular dysfunction and atherosclerosis [27–30, 42, 43]. Most T2DM patients are obese and have elevated plasma FFA levels [102, 103]. Moreover, high-fat diets might induce metabolic dysfunction and inflammation through the release of FFA through lipolysis and proinflammatory cytokines through downstream signaling [104, 105].

FFAs have been suggested to induce chronic low-grade inflammation, activate the innate immune system, and cause deleterious effects on vascular cells and other tissues through inflammatory processes. The results obtained in the present study demonstrated that hyperglycemia in diabetes exacerbates* in vitro* inflammatory responses in PBMNCs stimulated with high levels of SFA (palmitate). Furthermore, the results suggest that the endothelium levels of NO could be regulated through oxidative stress and high levels of triglycerides are correlated with oxidative stress and proinflammatory cytokine secretion in T2DM patients. Endothelial dysfunction is associated with several pathophysiological conditions in diabetes [56]. Combined therapy targeting the intracellular mechanisms underlying metabolic alterations leading to endothelial dysfunction is an important issue in the prevention of vascular complications associated with diabetes. The simultaneous control of hyperglycemia and hypertriglyceridemia is necessary to ameliorate the progression to diabetic vasculopathy.

## Figures and Tables

**Table 1:** Clinical and biochemical characteristics of the studied population.

Parameters	T2DM (*n* = 29)	ND (*n* = 16)	*P*
Female/Male ratio	19/10	11/5	NA
Age (years)	58.3 ± 9.0	57.1 ± 10.0	ns
Body mass index (kg/m^2^)	30.8 ± 9.8	24.6 ± 4.1	<0.05
Disease duration (years)	6.7 ± 6.4	NA	NA
Systolic pressure (mmHg)	127.9 ± 14.5	122.3 ± 15.9	ns
Diastolic pressure (mmHg)	86.6 ± 8.6	88.9 ± 7.9	ns
Fasting glucose (mg/dL)	147.0 ± 40.7	89.0 ± 9.0	<0.05
Glycated hemoglobin (%)	8.1 ± 1.1	5.3 ± 0.2	<0.05
Total cholesterol (mg/dL)	191.6 ± 65.7	160.7 ± 20.0	ns
Low density lipoprotein (mg/dL)	115.3 ± 39.7	104.5 ± 32.6	ns
High density lipoprotein (mg/dL)	45.6 ± 10.6	50.2 ± 14.0	ns
Triglycerides (mg/dL)	142.0 ± 51.0	108.6 ± 37.7	<0.05

Data as means ± SD.

NA: not applicable; ns: not significant.

Significant differences between the groups were determined using Student's *t*-test (*P* < 0.05).

**Table 2:** Plasma levels of oxidative stress biomarkers and proinflammatory cytokines.

Parameter	T2DM (*n* = 29)	ND (*n* = 16)	*P*
Nitric Oxide (*μ*M)	53.5 ± 12.9	51.13 ± 8.7	ns
MDA (*μ*M)	14.5 ± 3.5	8.7 ± 3.3	<0.05
IL-6 (pg/mL)	119.1 ± 23.3	97.6 ± 13.5	<0.05
TNF-alpha (pg/mL)	78.7 ± 32.7	58.5 ± 29.5	<0.05

Data as means ± SD.

ns: not significant.

Significant differences between the groups were determined using Student's *t*-test (*P* < 0.05).

## References

[1] Bergman RN, Ader M (2000). Free fatty acids and pathogenesis of type 2 diabetes mellitus. *Trends in Endocrinology and Metabolism*.

[2] Boden G (1997). Role of fatty acids in the pathogenesis of insulin resistance and NIDDM. *Diabetes*.

[3] Stich V, Berlan M (2004). Physiological regulation of NEFA availability: lipolysis pathway. *Proceedings of the Nutrition Society*.

[4] Tsimikas S, Reaven PD (1998). The role of dietary fatty acids in lipoprotein oxidation and atherosclerosis. *Current Opinion in Lipidology*.

[5] Reaven GM (1988). Role of insulin resistance in human disease. *Diabetes*.

[6] Ulven T (2012). Short-chain free fatty acid receptors FFA2/GPR43 and FFA3/GPR41 as new potential therapeutic targets. *Frontiers in Endocrinology*.

[7] Vinolo MAR, Hirabara SM, Curi R (2012). G-protein-coupled receptors as fat sensors. *Current Opinion in Clinical Nutrition and Metabolic Care*.

[8] Bunn RC, Cockrell GE, Ou Y, Thrailkill KM, Lumpkin CK, Fowlkes JL (2010). Palmitate and insulin synergistically induce IL-6 expression in human monocytes. *Cardiovascular Diabetology*.

[9] Egan BM, Lu G, Greene EL (1999). Vascular effects of non-esterified fatty acids: implications for the cardiovascular risk factor cluster. *Prostaglandins Leukotrienes and Essential Fatty Acids*.

[10] Hennig B, Shasby DM, Spector AA (1985). Exposure to fatty acid increases human low density lipoprotein transfer across cultured endothelial monolayers. *Circulation Research*.

[11] Horrobin DF (1995). Abnormal membrane concentrations of 20 and 22-carbon essential fatty acids: a common link between risk factors and coronary and peripheral vascular disease?. *Prostaglandins Leukotrienes and Essential Fatty Acids*.

[12] Shulman GI (2000). Cellular mechanisms of insulin resistance. *Journal of Clinical Investigation*.

[13] Giugliano D, Ceriello A, Paolisso G (1996). Oxidative stress and diabetic vascular complications. *Diabetes Care*.

[14] Hatanaka E, Levada-Pires AC, Pithon-Curi TC, Curi R (2006). Systematic study on ROS production induced by oleic, linoleic, and *γ*-linolenic acids in human and rat neutrophils. *Free Radical Biology and Medicine*.

[15] Wenzel U, Nickel A, Daniel H (2005). Increased mitochondrial palmitoylcarnitine/carnitine countertransport by flavone causes oxidative stress and apoptosis in colon cancer cells. *Cellular and Molecular Life Sciences*.

[16] Dhaunsi GS, Kaur J, Alsaeid K, Turner RB, Bitar MS (2005). Very long chain fatty acids activate NADPH oxidase in human dermal fibroblasts. *Cell Biochemistry and Function*.

[17] Horani MH, Haas MJ, Mooradian AD (2006). Saturated, unsaturated, and trans-fatty acids modulate oxidative burst induced by high dextrose in human umbilical vein endothelial cells. *Nutrition*.

[18] Duval C, Cámara Y, Hondares E, Sibille B, Villarroya F (2007). Overexpression of mitochondrial uncoupling protein-3 does not decrease production of the reactive oxygen species, elevated by palmitate in skeletal muscle cells. *FEBS Letters*.

[19] Srivastava S, Chan C (2007). Hydrogen peroxide and hydroxyl radicals mediate palmitate-induced cytotoxicity to hepatoma cells: relation to mitochondrial permeability transition. *Free Radical Research*.

[20] Rachek LI, Musiyenko SI, LeDoux SP, Wilson GL (2007). Palmitate induced mitochondrial deoxyribonucleic acid damage and apoptosis in L6 rat skeletal muscle cells. *Endocrinology*.

[21] Dasu MR, Jialal I (2011). Free fatty acids in the presence of high glucose amplify monocyte inflammation via Toll-like receptors. *American Journal of Physiology*.

[22] Lambertucci RH, Hirabara SM, Silveira LDR, Levada-Pires AC, Curi R, Pithon-Curi TC (2008). Palmitate increases superoxide production through mitochondrial electron transport chain and NADPH oxidase activity in skeletal muscle cells. *Journal of Cellular Physiology*.

[23] Darley-Usmar V, Wiseman H, Halliwell B (1995). Nitric oxide and oxygen radicals: a question of balance. *FEBS Letters*.

[24] McGrowder D, Ragoobirsingh D, Brown P (2006). Acute effects of exogenous nitric oxide on glucose uptake in skeletal muscle of normoglycaemic and diabetic rats. *Medical Science Monitor*.

[25] Jackson MJ, Pye D, Palomero J (2007). The production of reactive oxygen and nitrogen species by skeletal muscle. *Journal of Applied Physiology*.

[26] Pattwell DM, McArdle A, Morgan JE, Patridge TA, Jackson MJ (2004). Release of reactive oxygen and nitrogen species from contracting skeletal muscle cells. *Free Radical Biology and Medicine*.

[27] Kim JK (2006). Fat uses a TOLL-road to connect inflammation and diabetes. *Cell Metabolism*.

[28] Martins de Lima T, Gorjão R, Hatanaka E (2007). Mechanisms by which fatty acids regulate leucocyte function. *Clinical Science*.

[29] Shi H, Kokoeva MV, Inouye K, Tzameli I, Yin H, Flier JS (2006). TLR4 links innate immunity and fatty acid-induced insulin resistance. *Journal of Clinical Investigation*.

[30] Stoddart LA, Smith NJ, Milligan G (2008). International union of pharmacology. LXXI. Free fatty acid receptors FFA1, -2, and -3: pharmacology and pathophysiological functions. *Pharmacological Reviews*.

[31] Kolb H, Mandrup-Poulsen T (2005). An immune origin of type 2 diabetes?. *Diabetologia*.

[32] Tripathy D, Mohanty P, Dhindsa S (2003). Elevation of free fatty acids induces inflammation and impairs vascular reactivity in healthy subjects. *Diabetes*.

[33] Azekoshi Y, Yasu T, Watanabe S (2010). Free fatty acid causes leukocyte activation and resultant endothelial dysfunction through enhanced angiotensin II production in mononuclear and polymorphonuclear cells. *Hypertension*.

[34] Morohoshi M, Fujisawa K, Uchimura I, Numano F (1996). Glucose-dependent interleukin 6 and tumor necrosis factor production by human peripheral blood monocytes in vitro. *Diabetes*.

[35] Dasu MR, Devaraj S, Zhao L, Hwang DH, Jialal I (2008). High glucose induces toll-like receptor expression in human monocytes mechanism of activation. *Diabetes*.

[36] Inoguchi T, Li P, Umeda F (2000). High glucose level and free fatty acid stimulate reactive oxygen species production through protein kinase C-dependent activation of NAD(P)H oxidase in cultured vascular cells. *Diabetes*.

[37] Håversen L, Danielsson KN, Fogelstrand L, Wiklund O (2009). Induction of proinflammatory cytokines by long-chain saturated fatty acids in human macrophages. *Atherosclerosis*.

[38] Morigi M, Angioletti S, Imberti B (1998). Leukocyte-endothelial interaction is augmented by high glucose concentrations and hyperglycemia in a NF-kB-dependent fashion. *Journal of Clinical Investigation*.

[39] Guha M, Bai W, Nadler JL, Natarajan R (2000). Molecular mechanisms of tumor necrosis factor *α* gene expression in monocytic cells via hyperglycemia-induced oxidant stress-dependent and -independent pathways. *Journal of Biological Chemistry*.

[40] Laine PS, Schwartz EA, Wang Y (2007). Palmitic acid induces IP-10 expression in human macrophages via NF-*κ*B activation. *Biochemical and Biophysical Research Communications*.

[41] Lee JY, Ye J, Gao Z (2003). Reciprocal modulation of toll-like receptor-4 signaling pathways involving MyD88 and phosphatidylinositol 3-kinase/AKT by saturated and polyunsaturated fatty acids. *Journal of Biological Chemistry*.

[42] Maloney E, Sweet IR, Hockenbery DM (2009). Activation of NF-*κ*B by palmitate in endothelial cells: a key role for NADPH oxidase-derived superoxide in response to TLR4 activation. *Arteriosclerosis, Thrombosis, and Vascular Biology*.

[43] Covington DK, Briscoe CA, Brown AJ, Jayawickreme CK (2006). The G-protein-coupled receptor 40 family (GPR40-GPR43) and its role in nutrient sensing. *Biochemical Society Transactions*.

[44] Grundy SM, Denke MA (1990). Dietary influences on serum lipids and lipoproteins. *Journal of Lipid Research*.

[45] Richieri GV, Kleinfeld AM (1995). Unbound free fatty acid levels in human serum. *Journal of Lipid Research*.

[46] (2010). Diagnosis and classification of diabetes mellitus. *Diabetes Care*.

[47] Cousin SP, Hügl SR, Wrede CE, Kajio H, Myers MG, Rhodes CJ (2001). Free fatty acid-induced inhibition of glucose and insulin-like growth factor I-induced deoxyribonucleic acid synthesis in the pancreatic *β*-cell line INS-1. *Endocrinology*.

[48] Bicalho HMS, Gontijo CM, Nogueira-Machado JA (1981). A simple technique for simultaneous human leukocytes separation. *Journal of Immunological Methods*.

[49] Creager MA, Lüscher TF, Cosentino F, Beckman JA (2003). Diabetes and vascular disease. Pathophysiology, clinical consequences, and medical therapy: part I. *Circulation*.

[50] Lamharzi N, Renard CB, Kramer F (2004). Hyperlipidemia in concert with hyperglycemia stimulates the proliferation of macrophages in atherosclerotic lesions: potential role of glucose-oxidized LDL. *Diabetes*.

[51] Okuyama R, Fujiwara T, Ohsumi J (2003). High glucose potentiates palmitate-induced NO-mediated cytotoxicity through generation of superoxide in clonal *β*-cell HIT-T15. *FEBS Letters*.

[52] Boden G, She P, Mozzoli M (2005). Free fatty acids produce insulin resistance and activate the proinflammatory nuclear factor-*κ*b pathway in rat liver. *Diabetes*.

[53] Gao Z, Zhang X, Zuberi A (2004). Inhibition of insulin sensitivity by free fatty acids requires activation of multiple serine kinases in 3T3-L1 adipocytes. *Molecular Endocrinology*.

[54] Jové M, Planavila A, Sánchez RM, Merlos M, Laguna JC, Vázquez-Carrera M (2006). Palmitate induces tumor necrosis factor-*α* expression in C2C12 skeletal muscle cells by a mechanism involving protein kinase C and nuclear factor-*κ*B activation. *Endocrinology*.

[55] Takahashi HK, Cambiaghi TD, Luchessi AD (2012). Activation of survival and apoptotic signaling pathways in lymphocytes exposed to palmitic acid. *Journal of Cellular Physiology*.

[56] Sena CM, Pereira AM, Seica R (2013). Endothelial dysfunction—a major mediator of diabetic vascular disease. *Biochimica et Biophysica Acta*.

[57] Suganami T, Tanimoto-Koyama K, Nishida J (2007). Role of the Toll-like receptor 4/NF-*κ*B pathway in saturated fatty acid-induced inflammatory changes in the interaction between adipocytes and macrophages. *Arteriosclerosis, Thrombosis, and Vascular Biology*.

[58] Baldwin AS (1996). The NF-kappa B and I kappa B proteins: new discoveries and insights. *Annual Review of Immunology*.

[59] Wellen KE, Hotamisligil GS (2005). Inflammation, stress, and diabetes. *Journal of Clinical Investigation*.

[60] Pahl HL (1999). Activators and target genes of Rel/NF-*κ*B transcription factors. *Oncogene*.

[61] Kubes P (1993). Polymorphonuclear leukocyte-endothelium interactions: a role for pro-inflammatory and anti-inflammatory molecules. *Canadian Journal of Physiology and Pharmacology*.

[62] MacMicking J, Xie Q-W, Nathan C (1997). Nitric oxide and macrophage function. *Annual Review of Immunology*.

[63] Marletta MA, Hurshman AR, Rusche KM (1998). Catalysis by nitric oxide synthase. *Current Opinion in Chemical Biology*.

[64] Grozdanovic Z (2001). NO message from muscle. *Microscopy Research and Technique*.

[65] Moncada S, Palmer RMJ, Higgs EA (1991). Nitric oxide: physiology, pathophysiology, and pharmacology. *Pharmacological Reviews*.

[66] Nathan C, Xie Q-W (1994). Nitric oxide synthases: roles, tolls, and controls. *Cell*.

[67] Lambertucci RH, Leandro CG, Vinolo MA (2012). The effects of palmitic acid on nitric oxide production by rat skeletal muscle: mechanism via superoxide and iNOS activation. *Cellular Physiology and Biochemistry*.

[68] Esenabhalu VE, Schaeffer G, Graier WF (2003). Free fatty acid overload attenuates Ca^2+^ signaling and NO production in endothelial cells. *Antioxidants and Redox Signaling*.

[69] Li H, Li H, Bao Y, Zhang X, Yu Y (2011). Free fatty acids induce endothelial dysfunction and activate protein kinase C and nuclear factor-*κ*B pathway in rat aorta. *International Journal of Cardiology*.

[70] Tang Y, Li G (2011). Chronic exposure to high fatty acids impedes receptor agonist-induced nitric oxide production and increments of cytosolic Ca^2+^ levels in endothelial cells. *Journal of Molecular Endocrinology*.

[71] Kim F, Tysseling KA, Rice J (2005). Free fatty acid impairment of nitric oxide production in endothelial cells is mediated by IKK*β*. *Arteriosclerosis, Thrombosis, and Vascular Biology*.

[72] Meleth AD, Agrón E, Chan C-C (2005). Serum inflammatory markers in diabetic retinopathy. *Investigative Ophthalmology and Visual Science*.

[73] Celebiler Cavusoglu A, Bilgili S, Alaluf A (2007). Vascular endothelial growth factor level in the serum of diabetic patients with retinopathy. *Annals of Ophthalmology*.

[74] Ozturk BT, Bozkurt B, Kerimoglu H, Okka M, Kamis U, Gunduz K (2009). Effect of serum cytokines and VEGF levels on diabetic retinopathy and macular thickness. *Molecular Vision*.

[75] Kalousová M, Zima T, Tesař V, Dusilová-Sulková S, Škrha J (2005). Advanced glycoxidation end products in chronic diseases—clinical chemistry and genetic background. *Mutation Research*.

[76] Alexandraki K, Piperi C, Kalofoutis C, Singh J, Alaveras A, Kalofoutis A (2006). Inflammatory process in type 2 diabetes: the role of cytokines. *Annals of the New York Academy of Sciences*.

[77] Alexandraki KI, Piperi C, Ziakas PD (2008). Cytokine secretion in long-standing diabetes mellitus type 1 and 2: associations with low-grade systemic inflammation. *Journal of Clinical Immunology*.

[78] Pickup JC (2004). Inflammation and activated innate immunity in the pathogenesis of type 2 diabletes. *Diabetes Care*.

[79] Pickup JC, Chusney GD, Thomas SM, Burt D (2000). Plasma interleukin-6, tumour necrosis factor *α* and blood cytokine production in type 2 diabetes. *Life Sciences*.

[80] Pickup JC, Crook MA (1998). Is type II diabetes mellitus a disease of the innate immune system?. *Diabetologia*.

[81] Ceriello A (2003). New insights on oxidative stress and diabetic complications may lead to a “causal” antioxidant therapy. *Diabetes Care*.

[82] Dandona P, Aljada A, Bandyopadhyay A (2004). Inflammation: the link between insulin resistance, obesity and diabetes. *Trends in Immunology*.

[83] Dandona P, Aljada A, Chaudhuri A, Bandyopadhyay A (2003). The potential influence of inflammation and insulin resistance on the pathogenesis and treatment of atherosclerosis-related complications in type 2 diabetes. *Journal of Clinical Endocrinology and Metabolism*.

[84] Donath MY, Shoelson SE (2011). Type 2 diabetes as an inflammatory disease. *Nature Reviews Immunology*.

[85] Esper R, Vilariño J, Machado R, Paragano A (2008). Endothelial dysfunction in normal and abnormal glucose metabolism. *Advances in Cardiology*.

[86] Hatanaka E, Monteagudo PT, Marrocos MSM, Campa A (2006). Neutrophils and monocytes as potentially important sources of proinflammatory cytokines in diabetes. *Clinical and Experimental Immunology*.

[87] Herder C, Brunner EJ, Rathmann W (2009). Elevated levels of the anti-inflammatory interleukin-1 receptor antagonist precede the onset of type 2 diabetes: the whitehall II study. *Diabetes Care*.

[88] Herder C, Illig T, Rathmann W (2005). Inflammation and type 2 diabetes: results from KORA Augsburg. *Gesundheitswesen*.

[89] Mirza S, Hossain M, Mathews C (2012). Type 2-diabetes is associated with elevated levels of TNF-alpha, IL-6 and adiponectin and low levels of leptin in a population of Mexican Americans: a cross-sectional study. *Cytokine*.

[90] Rösen P, Nawroth PP, King G, Möller W, Tritschler H-J, Packer L (2001). The role of oxidative stress in the onset and progression of diabetes and its complications: a summary of a congress series sponsored by UNESCO-MCBN, the American diabetes association and the German diabetes society. *Diabetes/Metabolism Research and Reviews*.

[91] Shoelson SE, Lee J, Goldfine AB (2006). Inflammation and insulin resistance. *Journal of Clinical Investigation*.

[92] Spranger J, Kroke A, Möhlig M (2003). Inflammatory cytokines and the risk to develop type 2 diabetes: results of the prospective population-based European Prospective Investigation into Cancer and Nutrition (EPIC)-Potsdam study. *Diabetes*.

[93] Syed MA, Barinas-Mitchell E, Pietropaolo SL (2002). Is type 2 diabetes a chronic inflammatory/autoimmune disease?. *Diabetes, Nutrition and Metabolism*.

[94] Beckman JA, Creager MA, Libby P (2002). Diabetes and atherosclerosis epidemiology, pathophysiology, and management. *Journal of the American Medical Association*.

[95] Nesto RW (2004). Correlation between cardiovascular disease and diabetes mellitus: current concepts. *American Journal of Medicine*.

[96] McAndrew J, Patel RP, Jo H (1997). The interplay of nitric oxide and peroxynitrite with signal transduction pathways: implications for disease. *Seminars in Perinatology*.

[97] Bartesaghi S, Ferrer-Sueta G, Peluffo G (2007). Protein tyrosine nitration in hydrophilic and hydrophobic environments. *Amino Acids*.

[98] Radi R (2004). Nitric oxide, oxidants, and protein tyrosine nitration. *Proceedings of the National Academy of Sciences of the United States of America*.

[99] Large V, Arner P (1998). Regulation of lipolysis in humans. Pathophysiological modulation in obesity diabetes, and hyperlipidaemia. *Diabetes and Metabolism*.

[100] Chen Y-DI, Golay A, Swislocki ALM, Reaven GM (1987). Resistance to insulin suppression of plasma free fatty acid concentrations and insulin stimulation of glucose uptake in noninsulin-dependent diabetes mellitus. *Journal of Clinical Endocrinology and Metabolism*.

[101] Unger RH (1995). Lipotoxicity in the pathogenesis of obesity-dependent NIDDM: genetic and clinical implications. *Diabetes*.

[102] Reaven GM, Hollenbeck C, Jeng C-Y, Wu MS, Chen Y-DI (1988). Measurement of plasma glucose, free fatty acid, lactate, and insulin for 24 h in patients with NIDDM. *Diabetes*.

[103] Boden G (1996). Fatty acids and insulin resistance. *Diabetes Care*.

[104] Guilherme A, Virbasius JV, Puri V, Czech MP (2008). Adipocyte dysfunctions linking obesity to insulin resistance and type 2 diabetes. *Nature Reviews Molecular Cell Biology*.

[105] Bays H, Mandarino L, DeFronzo RA (2004). Role of the adipocyte, free fatty acids, and ectopic fat in pathogenesis of type 2 diabetes mellitus: peroxisomal proliferator-activated receptor agonists provide a rational therapeutic approach. *Journal of Clinical Endocrinology and Metabolism*.

